# Polymicrobial Pseudomonas Plus Candida Parapsilosis Endocarditis in an Injection Drug User: Considerations for Diagnosis and Management

**DOI:** 10.7759/cureus.13507

**Published:** 2021-02-23

**Authors:** Maninder Kaur, Parminder Virdi, Ramanjit Kaur, Aiden Abidov, Diane L Levine

**Affiliations:** 1 Internal Medicine, Wayne State University Detroit Medical Center, Detroit, USA; 2 Cardiology, John D. Dingell Veterans Affairs Medical Center, Detroit, USA

**Keywords:** fungal endocarditis, pseudomonas aeruginosa and candida parapsilosis, injection drug use, cd4 lymphocytopenia

## Abstract

Fungal endocarditis (FE) is a potentially lethal condition and its diagnosis can be challenging due to the low yield from blood cultures. FE should be suspected in patients with associated risk factors despite the identification of positive bacterial blood cultures. The common risk factors for FE discussed in the literature are total parenteral nutrition, immune suppression, prior antimicrobial therapy, intravenous drug addiction, and cardiac surgery. In this report, we discuss a patient who had positive blood cultures for *Pseudomonas* but was found to have *Candida parapsilosis *on valve culture. Physicians need to maintain a high index of suspicion for co-infective endocarditis in this patient population.

## Introduction

Fungal endocarditis (FE) is a rare entity, which accounts for only 2-4% of all cases of infective endocarditis (IE) [1]. Risk factors for FE include vascular lines, cardiac surgery, immunocompromised state, and injection drug use (IDU) [2]. FE is associated with a high mortality rate of 30-50%. The diagnosis of FE can be challenging as only 50% of cases tend to have positive blood cultures for fungus [3].

## Case presentation

A 59-year-old man with active IDU presented with a two-day history of fever, generalized malaise, and decreased appetite. He denied shortness of breath, palpitations, chest pain, sweating weakness, seizure, sensory loss, pain in the abdomen, or hematuria. Past medical history was remarkable for hepatitis C (HCV). The patient had idiopathic CD4 lymphocytopenia and had repeatedly tested negative for HIV and other causes of CD4 cell depletion. He was an active injection drug user with his latest injection of heroin occurring on the day of admission.

On examination, he appeared acutely ill. His vitals demonstrated a temperature of 100.7 ℉, blood pressure of 98/65 mmHg, respiratory rate of 24 breaths/minute, and heart rate of 103 beats/minute. The examination was notable for healed scars on both forearms and clubbing of his nails but was negative for rash, Janeway lesions, Osler nodes, cardiac murmur, splenomegaly, or joint abnormalities. Fundoscopic eye examination was normal.

Laboratory findings revealed anemia with hemoglobin of 8 gm/dl, a WBC count of 5.8 x 10^9^/L, a CD4 count of 190, and platelets of 63 x 10^9^/L. The patient also had hyponatremia and hypokalemia with a serum sodium level of 131 mmol/l and serum potassium of 3.2 mmol/l. His blood urea nitrogen was 33 mmol/l and serum creatinine was 1.8 mg/dl (baseline creatinine of 1 mg/dl). Urine microscopy was positive for 10-20 RBCs and 2+ protein in the urine. The patient's chest X-ray was normal. EKG showed sinus tachycardia without heart block. The patient was started on intravenous vancomycin and cefepime for empiric treatment of sepsis.

**Figure 1:**
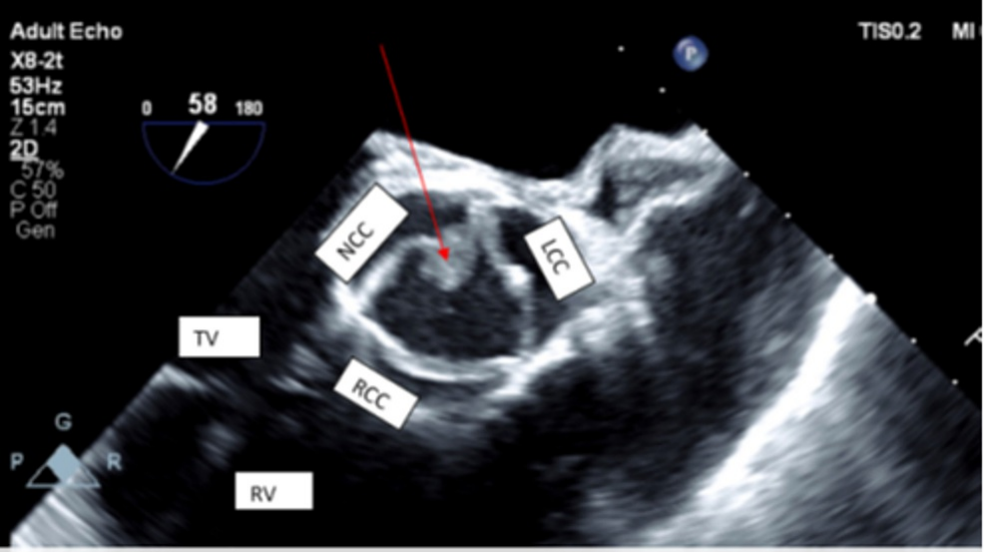
Still frame of the short axis of the aortic valve in systole The image shows a thickened, trileaflet aortic valve. The red arrow points to large vegetation noted on the non-coronary cusp and left coronary cusp of the aortic valve NCC: non-coronary cusp; LCC: left coronary cusp; RCC: right coronary cusp; TV: tricuspid valve; RV: right ventricle

**Figure 2:**
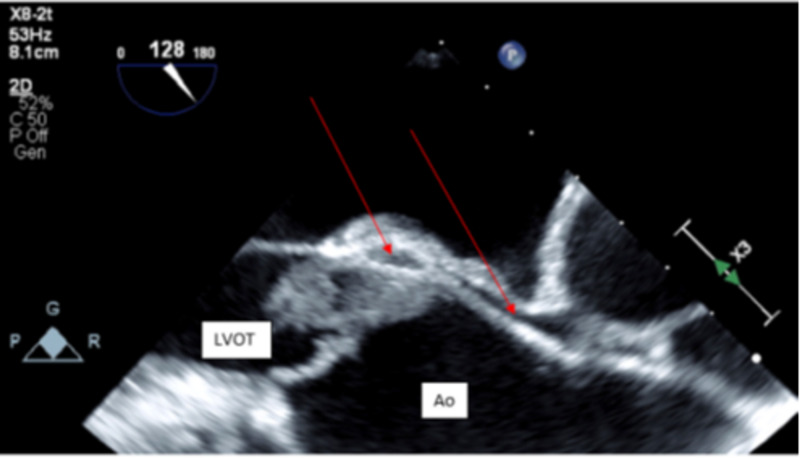
Still frame of apical 3 chamber with zoom on aortic root and ascending aorta The red arrow points to peri-valvular echolucent pockets consistent with an aortic valve abscess with extension into the aortic root, proximal ascending aorta, and inter-atrial septum Ao: ascending aorta; LVOT: left ventricle outflow tract

At 24 hours, two sets of blood cultures returned positive for *Pseudomonas aeruginosa* sensitive to cefepime, gentamicin, ciprofloxacin, imipenem, and piperacillin/tazobactam and tobramycin. On day three of admission, a transthoracic echocardiogram (TTE) was performed and showed possible vegetation on the aortic valve. This was confirmed by a transesophageal echocardiogram (TEE), which demonstrated large mobile vegetation on the non-coronary cusp protruding into the left ventricle outflow tract with each systole suggestive of an aortic root abscess (Figure 1, Figure 2).

The patient underwent urgent cardiothoracic surgery with placement of a bioprosthetic aortic valve and patch repair of aortic root abscess. Based on blood culture results, his antibiotic regimen was changed to cefepime and ciprofloxacin with a plan for six weeks of therapy. On day seven, the vegetation culture became positive for *Candida parapsilosis* and *Pseudomonas aeruginosa*. Intravenous liposomal amphotericin B and intravenous echinocandin were added to his regimen. Later, treatment was deescalated to cefepime and echinocandin. The patient completed six weeks of therapy and was found to be doing well three months after presentation.

## Discussion

IE is an inflammatory and proliferative disease of the endocardium that mainly affects the heart valves [4]. FE is a very rare entity and is seen in only 2% of all endocarditis cases; however, it is often a lethal condition. *Candida* and *Aspergillus* species are the two most commonly implicated pathogenic fungi in this condition.

In the United States, the rates of IE associated with IDU are on the rise, likely due to the increased opioid use associated with the ongoing opioid epidemic. The use of heroin nearly doubled between 2006 and 2013 [5]. There was an increase in IDU-related IE hospitalizations from 6-8% between 2000 and 2008 to 12% in 2013. Between 2007 and 2017, hospitalizations in North Carolina for IDU-associated IE increased approximately 12-fold, from 0.92 to 10.95 per 100,000, translating into 2,600 hospitalizations for IE during this period [6]. This was associated with a steady increase in surgery for IDU-associated IE. The epidemiology of IDU-related IE has mostly been associated with younger patients, a higher proportion of whites, and it has shown a similar distribution between men and women [7].

The pathogenesis of IE in IDU involves the injection of particulate matter and the diluent used for injection along with the illicit drug. This mixture can cause endothelial damage to the tricuspid valves. Heroin can increase pulmonary artery pressure, which creates more turbulence across the tricuspid valve, allowing particular matter into the pulmonary circulation [8]. Particulate matter of up to 8-10 mm in size can cross pulmonary capillaries and, in turn, damage the endothelium of the mitral or aortic valves.

FE is still an infrequent entity comprising only 2-4% of IE. Blood cultures are negative in half of the cases, thereby making an early diagnosis of the condition challenging. Total parenteral nutrition, immune suppression, prior antimicrobial therapy, intravenous drug addiction, and cardiac surgery are the known risk factors for developing FE [9]. *Candida* species are the most common cause of FE, accounting for 50% of cases of FE. *Candida albicans* is seen in 25% of cases of FE. Non-*albicans* species including *Candida parapsilosis*, *Candida tropicalis*, and *Candida glabrata* are seen in 25% of total FE cases [1]. *Candida* infection is commonly found in IDU. In a review by Webb et al., lymphocytopenia was present in 22.1% of cases of FE. A potential risk factor discussed was antecedent lymphopenia due to immune suppression [10].

The diagnosis of FE is difficult due to the low yield from blood cultures. Histopathological examination is essential in culture-negative cases, which can be done from surgically excised valves, peripheral emboli, or systemic ulcers. Molecular methods, such as polymerase chain reaction (PCR) to detect fungal nuclear material like DNA in blood or in explanted valves, can expedite the diagnosis [11].

The most recent Clinical Practice Guideline for the Management of Candidiasis by the Infectious Diseases Society of America has noted that β-D-glucan detection has the potential to diagnose invasive candidiasis “days to weeks” before blood cultures become positive. Empiric treatment based on β-D-glucan has the potential advantage of shortening the time to initiation of antifungal therapy. Initiating empiric antifungal treatment may decrease the diagnostic sensitivity of β-D-glucan levels, but it correlates with responses to antifungal therapy [12].

In our patient, the diagnosis for FE was not suspected despite multiple factors associated with the fungal infection including IDU, large vegetation, valve abscess, and CD4 lymphocytopenia. In the setting of multiple risk factors, we suggest that identification of a bacterium should not decrease suspicion of FE. Molecular methods should be utilized to evaluate for the presence of fungi, including serum β-D-glucan and molecular methods. A decision to start empiric therapy may be considered in clinical situations where there is a strong suspicion for fungal infection and a positive β-D-glucan.

Initial anti-fungal treatment for *Candida* spp. endocarditis should be a high dose of echinocandin (caspofungin or micafungin or anidulafungin). Once the patient has stabilized and follow-up blood cultures are found to be negative, step-down therapy with oral fluconazole, if susceptible, can be utilized. In our patient, blood cultures were never positive for fungus but *Candida parapsilosis* was isolated from the valve and found to be sensitive to fluconazole. The infectious disease consultant, therefore, provided two weeks of dual therapy and then de-escalated to monotherapy with oral fluconazole.

## Conclusions

FE is a serious disease associated with high mortality rates. It should be suspected in patients with multiple risk factors despite a positive bacterial blood culture. This is the second reported case involving co-infection of *Pseudomonas aeruginosa* with *Candida parapsilosis*. Since the number of patients with risk factors for fungal infection is on the rise, we encourage physicians to be at high alert for bacterial and fungal co-infective endocarditis.
